# Antioxidant and Anti-Inflammatory Effect of Thai Shallot (*Allium ascalonicum* cv. *chiangmai*) and Cha-Miang (*Camellia sinensis* var. *assamica*) Extracts on Human Embryonic Kidney Cell Line (HEK293)

**DOI:** 10.3390/life16010141

**Published:** 2026-01-15

**Authors:** Jiraporn Laoung-on, Chalermpong Saenjum, Kongsak Boonyapranai, Sakaewan Ounjaijean

**Affiliations:** 1 Research Institute for Health Sciences, Chiang Mai University, Chiang Mai 50200, Thailand; jiraporn.l@cmu.ac.th (J.L.-o.); kongsak.b@cmu.ac.th (K.B.); 2 Office of Research Administration, Chiang Mai University, Chiang Mai 50200, Thailand; 3 Department of Pharmaceutical Sciences, Faculty of Pharmacy, Chiang Mai University, Chiang Mai 50200, Thailand; chalermpong.s@cmu.ac.th

**Keywords:** future food, functional ingredients, oxidative stress, inflammation, cytoprotective, flavonoids

## Abstract

Oxidative stress and inflammation are key drivers in the pathogenesis of various chronic diseases, including cardiovascular disease, neurodegenerative disorders, chronic kidney disease, and diabetes. This study evaluated the antioxidant and anti-inflammatory activities of SHE, CME, and FCME, all cultivated in northern Thailand. Human embryonic kidney cells (HEK293) were exposed to FeSO_4_ to induce oxidative stress and to LPS to stimulate inflammation. Cell viability was assessed using the MTT assay, while intracellular ROS production was measured using the DCFH-DA. Lipid peroxidation was quantified using the thiobarbituric acid reactive substances assay, and the interleukin-6 (IL-6) release was determined by ELISAs. All extracts demonstrated low cytotoxicity; however, cell death increased at 48 h compared to 24 h. At 200 µg/mL, SHE, CME, and FCME significantly reduced the H_2_O_2_-induced ROS generation, with the combined treatment of SHE and FCME producing a more pronounced reduction than the individual treatments. Furthermore, the combination of SHE and FCME markedly decreased malondialdehyde (MDA) and IL-6 levels compared with other groups. These findings suggest that shallot and cha-miang extracts, particularly in combination, exhibit promising antioxidant and anti-inflammatory properties in kidney cell models. This combination could therefore be explored as a nutraceutical strategy for the prevention and management of chronic kidney disease, in which oxidative stress and inflammation play pivotal roles. Overall, our finding highlight the potential of the combined use of SHE and FCME as a functional ingredients in the food and pharmaceutical industries.

## 1. Introduction

The inability of biological mechanisms to effectively neutralize free radicals causes oxidative stress and subsequent cellular damage. An excessive accumulation of free radicals may disrupt cellular integrity, ultimately leading to cell and tissue degeneration [1]. Oxidative damage is associated with increased aging and the onset of various diseases, including cancer, cardiovascular disease, neurological disorders, pulmonary diseases, and nephropathy [2]. Furthermore, oxidative stress significantly contributes to the development of various diseases, including hypertension, type 2 diabetes mellitus, Alzheimer’s disease, and systemic inflammatory response syndrome [3,4].

Nephropathy, often in the form of chronic kidney diseases, affects over 10% of the general population worldwide [5] and represents a severe microvascular complication of diabetes mellitus, posing substantial health risks and serving as the primary cause of end-stage renal failure [6]. This illness causes a significant pressure on global healthcare systems and strongly impacts patients’ quality of life [7]. Cell damage resulting from an imbalance between free radicals or reactive oxygen species (ROS) and antioxidant defense mechanisms is a major contributing factor to the development of diabetes and renal failure [8]. Lifestyle choices such as smoking, drinking alcohol, and the consumption of junk food contribute to an increase in oxidative stress in the human body, which adversely affects metabolism and may contribute to diabetes [9]. The consumption of substances with antioxidative and anti-inflammatory properties plays a crucial role in mitigating oxidative stress and will be beneficial in the prevention of diabetes mellitus and renal failure [10].

Conventional treatments for diabetes mellitus and its complications are typically costly and unavailable to many individuals in developing countries [11]. Consequently, natural antioxidants derived from plants have been extracted, purified, and isolated for use as food additives aimed at the prevention and control of diabetic nephropathy [12]. Moreover, individuals are increasingly prioritizing their consumption of functional foods for disease prevention [13]. Plants are abundant in secondary metabolites, such as phenolics, flavonoids, and other natural antioxidant substances, which are recognized for their therapeutic benefits [14]. Plant-based materials and herbal remedies have long been widely utilized to combat oxidative stress and prevent diabetes-associated renal damage, such as *Allium ascalonicum* [15] and *Camellia sinensis* [16].

Shallots (*Allium ascalonicum* L.) are a variety of red onion commonly cultivated in Southeast Asia [15]. They are extensively used as a culinary ingredient in Asian diets and have also been traditionally employed in folk medicine for their health-promoting features, including the alleviation of fever, digestive discomfort, and infections [15]. Shallots are abundant in organosulfur compounds, such as alliin and allicin, as well as the derivative of flavonoids and phenolic compounds [17]. Moreover, the skin of shallots is abundant in quercetin, which a powerful flavonoid antioxidant [18]. These compounds are strong natural antioxidants widely acknowledged for their diverse biological activities, including antibacterial, antiviral, antidiabetic, antioxidant, and anti-inflammation properties [19]. Therefore, shallot extract has received significant attention as a therapeutic for free radical management and for treating numerous oxidative-related diseases.

Tea (*Camellia sinensis*) is widely processed and consumed as a beverage around the world [20]. Cha-miang (*Camellia sinensis* var. *assamica*), a member of the Theaceae family, is predominantly cultivated in northern Thailand [21]. It exhibits a wide range of biological properties with phenolic compositions, including antioxidant, antimutagenic, and anticarcinogenic properties, as well as the inhibition of nitrosation and tumor development [22]. This plant contains several bioactive compounds, such as gallic acid, epigallocatechin, caffeine, catechin, epicatechin, and gallocatechin gallate, which have been reported to confer multiple pharmacological benefits, including protective effects against cancer, hypertension, inflammation, diabetes, obesity, hyperlipidemia, and cardiovascular disease [23,24]. Despite the popularity of shallots and tea as medicinal plants, limited research has been carried out on the Thai shallot and cha-miang.

Consequently, the present study aims to assess the extract of shallot (SHE), fresh cha-miang (CME), and fermented cha-miang (FCME) to examine their ability to manage diabetic nephropathy. This study evaluates the influence of plant extracts on antioxidant and anti-inflammatory effects in human embryonic kidney cells (HEK293) subjected to oxidative stress.

## 2. Materials and Methods

### 2.1. Chemicals and Reagents

All chemicals and standards used in this study were of analytical and cell culture grade, providing the highest level of purity appropriate for this research. Dulbecco’s Modified Eagle Medium (DMEM), fetal bovine serum (FBS), penicillin–streptomycin (pen-strep), and trypsin/EDTA were obtained from Gibco (Grand Island, NY, USA). Carboxy-2′,7′-dichloro-dihydro-fluorescein diacetate (DCFH-DA), 3-(4,5-dimethylthiazol-2-yl)-2,5-diphenyltetrazolium (MTT), Trolox (6-hydroxy-2,5,7,8-tetramethyl-2-carboxylic acid), and other chemicals were obtained from Sigma-Aldrich Co. (St. Louis, MO, USA).

### 2.2. Plant Materials and Extractions

Shallot (*Allium ascalonicum* cv. *Chiangmai*) bulbs that were pesticide-free were collected from the northern part of Thailand (Sanpatong District, Chiang Mai, Thailand) during March–April 2017. The plant materials were deposited and authenticated according to a previous study with a voucher number of SK-SH-CM01 [25]. The bulbs of shallots were prepared as fresh shallot juice according a prior report [15]. The sample was immediately dried by lyophilization, and 300 g of sample powder was reacted with 1 mg of *β*-glucosidase at 45 °C for 60 min (pH 5.5). After the enzyme reaction, the shallot extract (SHE) sample was re-dried by lyophilization and stored at −20 °C before examination.

The fresh cha-miang leaf extract (CME) and fermented cha-miang leaf extract (FCME) were harvested, deposited, and prepared according to a previous study with a specimen number of 00159 [22]. The dried extracts were kept at −20 °C until further experimental use.

### 2.3. Identification and Quantification of Quercetin and Quercetin Glycosides in SHE by HPLC

The chromatographic profiles of quercetin and quercetin glycosides in SHE were analyzed using reverse-phase high-performance liquid chromatography (HPLC). An analysis was performed using a Shimadzu SIL-20AC Prominence Autosampler HPLC system (Shimadzu, Tokyo, Japan) outfitted with a multi-wavelength detector, according to the methodology established in a previous investigation [26]. The separation was carried out on a Inertsil^®^ ODS-3 (GL Sciences Inc., Torrance, CA, USA) endcapped column (250 mm × 4.1 mm, 5 µm pore size). The chromatographic analysis employed a mobile phase composed of solvent A (5% formic acid in water) and solvent B (methanol). Shallot samples (20 µL) were injected for analysis and assessed at a wavelength of 360 nm. Compound identification was achieved by comparing the retention times and UV-Vis spectral characteristics of the eluted peaks with reference standards.

However, the identification and quantification of the bioactive compounds in CME and FCME were performed and demonstrated in a previous study [23].

### 2.4. Cell Culture

Human embryonic kidney cells (HEK293) were obtained from the American Type Culture Collection (Manassas, VA, USA) and maintained under standard cell culture conditions. The HEK293 cells were cultured in Dulbecco’s Modified Eagle’s Medium (DMEM) supplemented with 10% fetal bovine serum (FBS) and 100 IU/mL of pen–strep at 37 °C in a humidified atmosphere of 5% CO_2_. After the cells achieved 70–80% confluence, the cells were harvested and plated for subsequent passages or for the treatments.

### 2.5. Experimental Design

HEK293 cells were placed into a 96-well microplate overnight at a density of 5 × 10^3^ cells/well in DMEM for 24 h. Eight treatments were prepared as followed.

- Treatment I (Control): HEK293 cells in this group were added to normal DMEM.
- Treatment II (200 SHE): HEK293 cells were treated with 200 µg/mL of SHE.
- Treatment III (200 CME): HEK293 cells were treated with 200 µg/mL of CME.
- Treatment IV (200 FCME): HEK293 cells were treated with 200 µg/mL of FCME.
- Treatment V (100 SHE + CME): HEK293 cells were treated with a solution of 100 µg/mL of SHE and CME.
- Treatment VI (100 SHE + FCME): HEK293 cells were treated with a mixture of 100 µg/mL of SHE and FCME.
- Treatment VII (200 SHE + CME): HEK293 cells were treated with a mixture of 200 µg/mL of SHE and CME.
- Treatment VIII (200 SHE + FCME): HEK293 cells were treated with a mixture of 200 µg/mL of SHE and FCME.

All treatments were incubated at 37 °C in a humidified atmosphere of 5% CO_2_ for 24 and 48 h.

### 2.6. Cytotoxicity Assay

The cytotoxicity of SHE, CME, and FCME in HEK293 cells was determined using the MTT assay. After 24 and 48 h of treatment, the cells were flushed twice with PBS and subsequently collected. Subsequently, 20 µL of MTT dye (5 mg/mL in PBS) was added to each well and incubated at 37 °C for 4 h. The medium was subsequently removed, and 100 µL of DMSO was added to each well to dissolve the formazan crystals. The absorbance was measured using a microplate reader (STECTROstar Nano, BMG LABTECH, Ortenberg, Germany) at λ 540/630 nm [27].

### 2.7. Determination of Intracellular Reactive Oxygen Species (ROS) Production

The antioxidant effect of SHE, CME, and FCME on intracellular ROS production in HEK293 cells under hydrogen peroxide (H_2_O_2_)-induced oxidative stress was evaluated by the DCFH-DA assay according to a previous study [15]. Trolox, a derivative of Vitamin E, at a concentration of 200 µg/mL was used as a positive control, whereas H_2_O_2_ at 125 µM was used to induce oxidative stress. HEK293 cells were placed in a 96-well plate at a final density of 1 × 10^4^ cells/well and incubated at 37 °C with 5% CO_2_ for 24 h. The medium was removed and washed twice with PBS. Then, H_2_O_2_ was added to each well, followed by various treatment solutions and Trolox. Control cells were prepared by adding a fresh medium and incubated again for a further 15 min. After incubation, 10 µL of DCFH-DA was added to each well and incubated at 37 °C with 5% CO_2_ for 30 min in dark conditions. Reactive oxygen species (ROS) generation was measured using fluorescence intensity, measured at 488 nm of excitation and 617 nm of emission by a microplate reader.

### 2.8. Determination of Lipid Peroxidation (LPO) Production

The impact of SHE, CME, and FCME on the reduction in oxidative damage to lipids of the plasma membranes was investigated. Ferrous ions from ferrous ammonium sulfate (FeAS) were catalyzed via a Haber–Weiss and Fenton reaction, leading to ROS-induced lipid peroxidation. HEK293 cells were placed in a 96-well plate at a final density of 1 × 10^6^ cells/well and treated with various treatment solutions or Trolox, with 5 mM FeAS, for 24 h at 37 °C in a 5% CO_2_ atmosphere. After incubation, cells were harvested with a cell scraper and rinsed with PBS. Cells were homogenized and centrifuged to obtain pellets, and the amounts of MDA were quantified using the thiobarbituric acid reactive substance (TBARS) assay. After to the experimental technique, the supernatant was analyzed using a microplate reader at a wavelength of 535 nm [15].

### 2.9. Determination of Cytokine Release Production

The anti-inflammatory efficacy of SHE, CME, and FCME in HEK293 cells was determined by measuring the release of the inflammatory cytokine interleukin-6 (IL-6). HEK293 cells were placed in a 6-well plate at a density of 1.5 × 10^6^ cells/well and incubated with various treatment solutions or 10 µg/mL of dexamethasone (DEX) as the anti-inflammatory agent for 24 h at 37 °C in a 5% CO_2_ atmosphere. The release levels of IL-6 into the culture media were measured using a Human IL-6 ELISA Kit (ab46027) from Abcam (Waltham, MA, USA), according to a previous study [15].

### 2.10. Statistical Analysis

The data is presented as the mean ± standard deviation (SD) from three replicated samples across three different experiments. The normal distribution was determined using the Shapiro–Wilk test. The statistical analysis of the mean values of all parameters was performed using a one-way ANOVA followed by Duncan’s test in SPSS 22.0 (Chicago, IL, USA) to analyze the differences between groups. All significance values were determined at *p* ≤ 0.05.

## 3. Results

### 3.1. The Chromatographic Profiles and Quantification of Quercetin and Quercetin Glycosides in SHE

**Figure 1:**
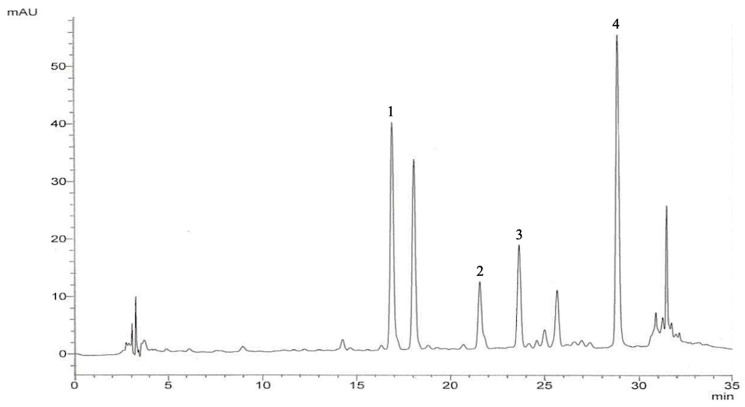
HPLC chromatograms of phenolic acids shown in the SHE column, Inertsil^®^ ODS-3; mobile phase, 5% formic acid in water and methanol; flow rate, 1 mL/min; and detection wavelength, 360 nm. Peak identification: peak 1, quercetin-3,4′-diglucoside; peak 2, quercetin-3-glucoside; peak 3, quercetin-4′-glucoside; and peak 4, quercetin aglycone.

The HPLC chromatogram of SHE presented quercetin-3,4′-diglucoside, quercetin-3-glucoside, quercetin-4′-glucoside, and quercetin aglycone (Figure 1), and the concentrations were 2.56 ± 0.11, 0.13 ± 0.11, 1.24 ± 0.01, and 4.78 ± 0.11 mg/g of plant extract, respectively.

### 3.2. Effect of SHE, CME, and FCME on HEK293 Cell Viability

**Figure 2:**
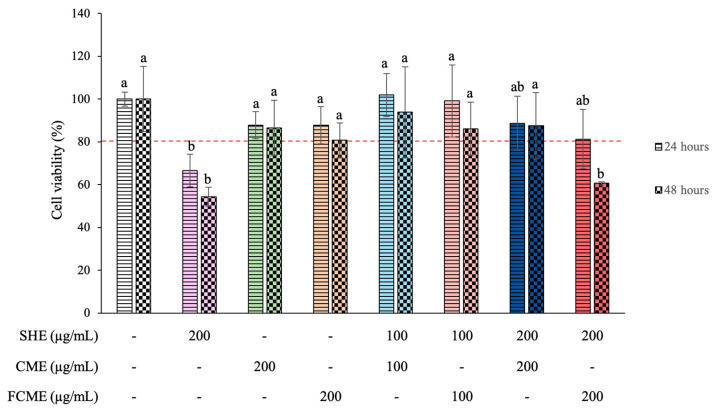
The percentage of HEK293 cell viability in controls treated with SHE, CME, FCME, and their combination. Different letters (a, b) indicate statistically significant differences among groups (*p* < 0.05). Data are presented as the mean ± standard deviation (error bar). All experiments were conducted in triplicate with three independent repetitions.

An MTT (3-[4,5-dimethylthiazol-2-yl]-2,5-diphenyl tetrazolium bromide) assay was performed to assess the cytotoxicity of various treatments in HEK293 cells for 24 and 48 h. The percentage of cell viability after the treatment with various extracts at different concentrations for 24 and 48 h was demonstrated (Figure 2). The control group, which received no treatment, demonstrated 100 percent cell viability. The group treated with 200 μg/mL of SHE displayed a significant reduction in cell viability at both 24 and 48 h, with a decline in viability at 48 h compared to 24 h. The treatment with CME and FCME at 200 μg/mL preserved a cell viability similar to the control group, indicating no presence of cytotoxicity. Furthermore, all groups treated with a combination treatment of 100 μg/mL of SHE, CME, and FCME demonstrated a cell viability above 80 percent. The group that received a combination of SHE and FCME at 200 μg/mL showed a significant reduction in cell viability after 48 h, suggesting potential cytotoxic effects with long-term exposure. However, the cell viability in all treatment groups surpassed 50 percent.

### 3.3. Effect of SHE, CME, and FCME on Intracellular ROS Production Under H_2_O_2_-Induced Oxidative Stress

**Figure 3:**
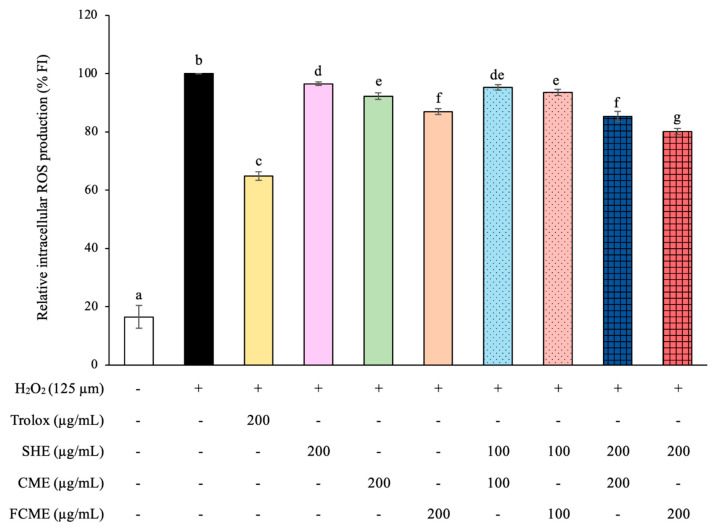
Relative intracellular ROS production in HEK293 cell treated with SHE, CME, FCME, and combination of these plant extracts during H_2_O_2_ exposure. Different letters (a–g) indicate statistically significant differences among groups (*p* < 0.05). Data are presented as the mean ± standard deviation (error bar). All experiments were conducted in triplicate with three independent repetitions.

We analyzed the relative intracellular ROS production of HEK293 cells treated with various treatments in the presence of H_2_O_2_-induced oxidative stress (Figure 3). The H_2_O_2_-induced group demonstrated a significant incline in the production of ROS when compared with the control group. Trolox, a vitamin E derivative, showed significantly reduced ROS production compared to the H_2_O_2_ group, indicating it had a protective effect against ROS production. All treatment groups also resulted in a significant reduction in ROS levels, especially the group treated with a combination of SHE and FCME at 200 μg/mL, which had the greatest potential to prevent ROS production, which is less effective than Trolox.

### 3.4. Effect of SHE, CME, and FCME on Intracellular LPO Production Under FeAS-Induced Lipid Peroxidation

**Figure 4:**
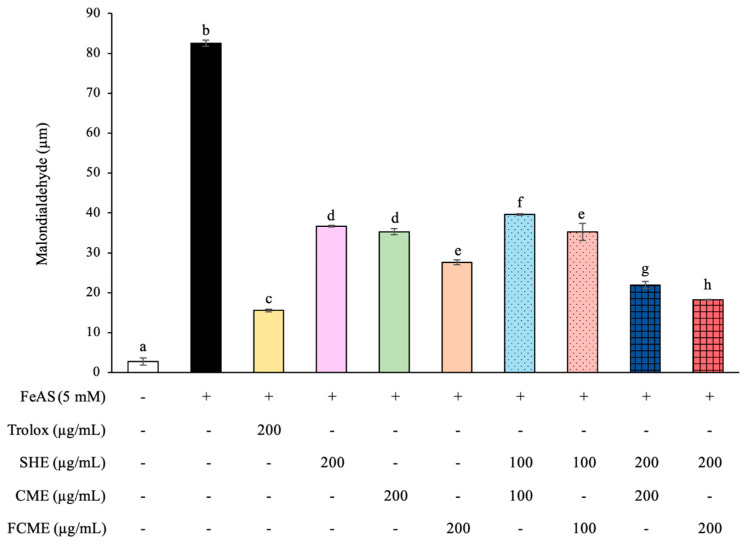
Levels of malondialdehyde in HEK293 cells treated with SHE, CME, FCME, and combination of these plant extracts during FeAS exposure. Different letters (a–h) indicate statistically significant differences among groups (*p* < 0.05). Data are presented as the mean ± standard deviation (error bar). All experiments were conducted in triplicate with three independent repetitions.

The result for the LPO concentration were demonstrated to be significantly inclined in the HEK293 cells induced with FeAS when compared with the control group (Figure 4). However, HEK293 cells treated with Trolox as a positive control had significantly reduced LPO production compared to the FeAS group, indicating that it had a protective effect against LPO production. All treatment groups demonstrated a significant reduction in LPO levels, especially the group that received the combination of SHE and FCME at 200 μg/mL, which had the most potential for preventing LPO production, which was less efficient than Trolox (Figure 4).

### 3.5. Effect of SHE, CME, and FCME on IL-6 Production Under LPS-Induced Inflammation

**Figure 5:**
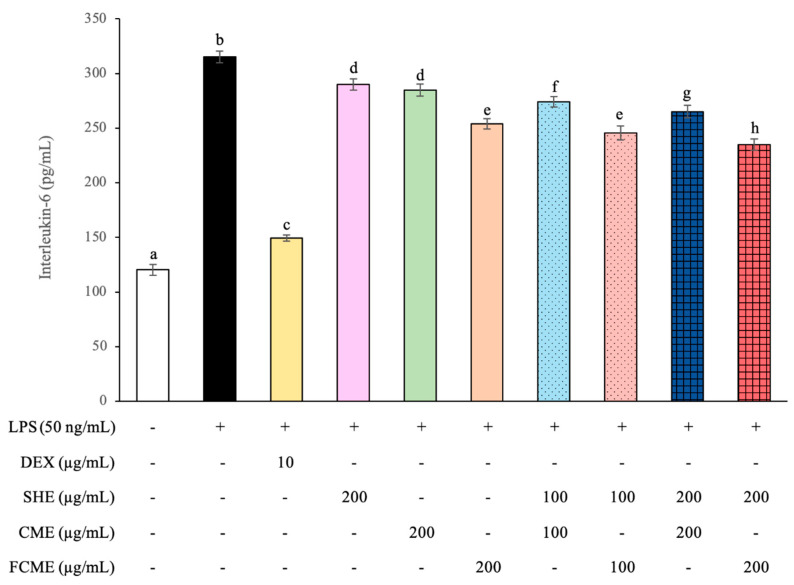
Levels of interleukin-6 release in response to HEK293 cell treated with SHE, CME, FCME, and combination of these plant extracts during LPS exposure. Different letters (a–h) indicate statistically significant differences among groups (*p* < 0.05). Data are presented as the mean ± standard deviation (error bar). All experiments were conducted in triplicate with three independent repetitions.

The effect of LPS on the IL-6 levels to reflect the inflammatory status in HEK293 cells has been shown Figure 5. The HEK293 cells induced with LPS significantly increased the IL-6 levels when compared with the normal control. However, HEK293 cells treated with dexamethasone (DEX, 10 μg/mL), a known anti-inflammatory agent, had significantly reduced IL-6 levels compared to the LPS group. All treatment groups demonstrated a significant reduction in IL-6 levels, especially the group that received the combination of SHE and FCME at 200 μg/mL, which had the most potential for preventing IL-6 production, which was less efficient than DEX.

## 4. Discussion

Shallots (*Allium ascalonicum* L.) are a red onion and well-known plant and essential ingredient in Asian cuisine and traditional medicine [15]. Shallots are abundant in flavonoids and phenolic compounds that are used for the health-promoting features, reducing fevers, flatulence, and infections [15,17]. The present study uses HPLC for qualitative and quantitative evaluations that examined quercetin-3,4′-diglucoside, quercetin-3-glucoside, quercetin-4′-glucoside, and quercetin aglycone. The result was similar to a previous report that shallots contained plenty of flavonoid contents [25]. Quercetin is a natural flavonoid abundantly present in shallots and demonstrates numerous health-promoting properties, including anti-inflammatory, antioxidant, antiviral, anti-allergic, and anticancer effects [28].

The MTT results show that most treatments, including combinations of SHE, CME, and FCME at 100 µg/mL, maintained a HEK293 cell viability above 80%, whereas SHE at 200 µg/mL, alone or in combination with FCME, significantly reduced viability, especially at 48 h, indicating potential cytotoxicity with prolonged exposure. Similarly, previous studies found that shallot extract reduced the cell viability of human vascular endothelial cell lines (EA. hy926) at 48 h compared to 24 h, but there was no significant difference when compared to the control [15]. Similarly, the previous studies found that cha-miang and fermented cha-miang extracts had no harmful effects in HEK298 cells [23]. The results indicate that the cytotoxic effects of the plant extracts are dependent on the exposure duration, similar to previous studies that demonstrated both the concentration- and time-dependent cytotoxicity of the plant extracts [29,30]. This indicates that some bioactive chemicals in SHE and FCME may induce cytotoxic effects at higher concentrations and with prolonged exposure. A previous study has similarly reported findings that demonstrate the significance of phytochemicals that impact cell viability responses [23,31].

Additionally, the H_2_O_2_-treated group demonstrated a significant increase in ROS production, which induced oxidative stress, leading to cellular damage and various diseases occurring [32,33]. However, Trolox used as positive control showed significantly reduced ROS production, indicating that it had protective effects against ROS production. Trolox is widely employed as a positive control due to its strong antioxidant capacity in reducing oxidative stress and protecting cells from damage [34,35]. All treatment groups also resulted in a significant decrease in ROS levels, especially the group treated with the combination of SHE and FCME at 200 μg/mL. Similarly, SHE, CME, and FCME had inhibited intracellular ROS production in endothelial cells [15], which is aligns with the result of a cell-free model that showed that CME and FCME had potential to scavenge DPPH and ABTS radicals [24]. A previous report found that CME and FCME contained an abundance of catechin and derivative compounds such as gallic acid, gallocatechin, epigallocatechin, caffeine, catechin, epicatechin, epigallocatechin gallate, gallocatechin gallate, and epicatechin gallate, which are flavonoids and phenolics [23]. Moreover, the SHE contained substantial total phenolic and total flavonoid contents [15,25]. Phenolics and flavonoids are the groups of phytochemical and polyphenolic compositions that are abundant in fruits and vegetables [36]. Phenolics and flavonoids had a strong natural antioxidant potential that reduces the oxidative stress of living cells [27,33,37].

The results for the LPO concentration were significantly increased in the HEK293 cells induced with FeAS, according to a previously used method for the FeAS-induced LPO of cell lines [15]. Normally, lipids are the macromolecules within living cells that compose the phospholipid bilayer of the cell membrane and are directly exposed to free radical particles, which are readily oxidized and display fluctuations [38]. The cell membrane includes plenty of polyunsaturated fatty acids (PUFAs), which control fluidity and permeability [39]. Large amounts of free radicals attract PUFAs, oxidizing lipids in the cell membrane and reducing its capacities [40]. All treatments markedly reduced LPO levels, with the SHE and FCME 200 µg/mL combination showing the strongest LPO-lowering effect, though still less effective than Trolox. They contain phytochemical compounds with unique capacities and reactions that have antioxidant actions at various concentrations [41]. Consequently, SHE, CME, and FCME may alter the cell membrane and LPO levels, and it is possible that they may impact cell membrane lipids, leading to permeability [42]. These mechanisms should be completely clarified in future studies. Therefore, the compounds of SHE, CME, and FCME also have a benefit for mitigating the cell damage of HEK293 cells treated with FeAS by scavenging free radicals.

This study used lipopolysaccharides (LPSs) to induce inflammation and produce inflammatory cytokines. The level of IL-6 released into culture media was used to measure kidney cell damage [43]. The LPS-treated group presented a significant increase in IL-6 levels when compared to the control, which correlates with previous studies indicating that LPS is a strong inducer of pro-inflammatory cytokines [44,45]. However, HEK293 cells treated with dexamethasone (DEX), which is an anti-inflammatory agent, had significantly reduced IL-6 levels, demonstrating its effectiveness in suppressing inflammation. The inhibition of IL-6 by DEX confirms its recognized function as an anti-inflammatory drug, supporting previous studies on its effectiveness in inflammation models [46]. All treatment groups demonstrated a significant reduction in IL-6 levels, especially the group that received the combination of SHE and FCME. This result indicated the availability of bioactive compounds that regulate cytokine production. Similarly, a previous study has demonstrated that polyphenols and flavonoids in plant extracts can suppress IL-6 expression by regulating NF-κB and MAPK signaling pathways [47,48].

Collectively, our data indicate that SHE, CME, and FCME, especially the combination of SHE and FCME at a dose of 200 µg/mL, could be used as an appropriate option for the management of diabetes-associated renal failure. Active compounds found in SHE, CME, and FCME, including phenolics and flavonoids, may facilitate a variety of health-related physiological functions, especially decreasing the incidence of renal disorders. This is partially attributable to their capacity to scavenge free radicals, prevent lipid peroxidation of the plasma membrane, and reduce inflammation, thereby protecting kidney cells from oxidative damage [49,50]. The extract’s cellular antioxidant activity in renal cells is clinically significant. Therefore, the primary treatment objective for diabetic renal disorders is to prevent oxidative stress-induced renal impairment.

## 5. Conclusions

In conclusion, SHE contained plenty of quercetin and quercetin glycosides, including quercetin-3,4′-diglucoside, quercetin-3-glucoside, quercetin-4′-glucoside, and quercetin aglycone. SHE, CME, and FCME, especially the combination of high doses of SHE and FCME, promotes significant antioxidant and anti-inflammatory properties in HEK293 cells, with substantial non-toxicity regarding viability during the initial 48 h of the culture. The findings indicate that these plant extracts may be beneficial as an effective regimen for managing disorders associated with oxidative stress. Our research reveals the potential of the combination of SHE and FCME as a functional ingredient in the food and pharmaceutical industries.

## Data Availability

The original contributions presented in this study are included in the article. Further inquiries can be directed at the corresponding author.

## References

[1] Chandimali N., Bak S.G., Park E.H., Lim H.-J., Won Y.-S., Kim E.-K., Park S.-I., Lee S.J. (2025). Free radicals and their impact on health and antioxidant defenses: A review. Cell Death Discov..

[2] Leyane T.S., Jere S.W., Houreld N.N. (2022). Oxidative stress in ageing and chronic degenerative pathologies: Molecular mechanisms involved in counteracting oxidative stress and chronic inflammation. Int. J. Mol. Sci..

[3] Halim M., Halim A. (2019). The effects of inflammation, aging and oxidative stress on the pathogenesis of diabetes mellitus (type 2 diabetes). Diabetes Metab. Syndr..

[4] Rojas-Gutierrez E., Muñoz-Arenas G., Treviño S., Espinosa B., Chavez R., Rojas K., Flores G., Díaz A., Guevara J. (2017). Alzheimer’s disease and metabolic syndrome: A link from oxidative stress and inflammation to neurodegeneration. Synapse.

[5] Kovesdy C.P. (2022). Epidemiology of chronic kidney disease: An update 2022. Kidney Int. Suppl..

[6] Adebayo-Gege G.I., Adegbola P.I., Adedayo L.D., Oyefabi A.M., Oyeyemi I.T., Olubukola O., Oke A.A., Okeke O.P., Abodunrin O.R., Akinsolu F.T. (2025). Prevalence of nephropathy among patients with diabetes mellitus in Africa: A systematic review and meta-analysis. Front. Clin. Diabetes Healthc..

[7] Francis A., Harhay M.N., Ong A.C., Tummalapalli S.L., Ortiz A., Fogo A.B., Fliser D., Roy-Chaudhury P., Fontana M., Nangaku M. (2024). Chronic kidney disease and the global public health agenda: An international consensus. Nat. Rev. Nephrol..

[8] Bhatti J.S., Sehrawat A., Mishra J., Sidhu I.S., Navik U., Khullar N., Kumar S., Bhatti G.K., Reddy P.H. (2022). Oxidative stress in the pathophysiology of type 2 diabetes and related complications: Current therapeutics strategies and future perspectives. Free Radic. Biol. Med..

[9] Man A.W., Li H., Xia N. (2020). Impact of lifestyles (diet and exercise) on vascular health: Oxidative stress and endothelial function. Oxid. Med. Cell Longev..

[10] Sahakyan G., Vejux A., Sahakyan N. (2022). The role of oxidative stress-mediated inflammation in the development of T2DM-induced diabetic nephropathy: Possible preventive action of tannins and other oligomeric polyphenols. Molecules.

[11] Misra A., Gopalan H., Jayawardena R., Hills A.P., Soares M., Reza-Albarrán A.A., Ramaiya K.L. (2019). Diabetes in developing countries. J. Diabetes.

[12] Lee O.Y.A., Wong A.N.N., Ho C.Y., Tse K.W., Chan A.Z., Leung G.P.-H., Kwan Y.W., Yeung M.H.Y. (2024). Potentials of natural antioxidants in reducing inflammation and oxidative stress in chronic kidney disease. Antioxidants.

[13] Christodoulou E., Laoutari S., Athanasiou F., Poutli E., Andreou D., Kourkoutas Y., Koutelidakis A.E. (2025). Bridging science and lifestyle: A feasibility study for developing a novel functional food to support well-being. Nutraceuticals.

[14] Sun W., Shahrajabian M.H. (2023). Therapeutic potential of phenolic compounds in medicinal plants—Natural health products for human health. Molecules.

[15] Ounjaijean S., Chachiyo S., Kulprachakarn K., Boonyapranai K., Srichairatanakool S., Rerkasem K. (2019). Antioxidant and anti-inflammatory protective properties of Thai shallot (*Allium ascalonicum* cv. *Chiangmai*) juice on human vascular endothelial cell lines (EA. hy926). Walailak J. Sci. Technol..

[16] Brimson J.M., Prasanth M.I., Kumaree K.K., Thitilertdecha P., Malar D.S., Tencomnao T., Prasansuklab A. (2022). Tea plant (*Camellia sinensis*): A current update on use in diabetes, obesity, and cardiovascular disease. Nutrients.

[17] Shahrajabian M.H., Wenli S., Cheng Q. (2020). Chinese onion, and shallot, originated in Asia, medicinal plants for healthy daily recipes. Not. Sci. Biol..

[18] Wiczkowski W., Romaszko J., Bucinski A., Szawara-Nowak D., Honke J., Zielinski H., Piskula M.K. (2008). Quercetin from Shallots (*Allium cepa* L. var. *aggregatum*) is more bioavailable than its glucosides. J. Nutr..

[19] Kaurinovic B., Vastag D. (2019). Flavonoids and phenolic acids as potential natural antioxidants. Antioxidants.

[20] Graham H.N. (1992). Green tea composition, consumption, and polyphenol chemistry. Prev. Med..

[21] Unban K., Khatthongngam N., Pattananandecha T., Saenjum C., Shetty K., Khanongnuch C. (2020). Microbial community dynamics during the non-filamentous fungi growth-based fermentation process of Miang, a traditional fermented tea of north Thailand and their product characterizations. Front. Microbiol..

[22] Unban K., Khatthongngam N., Shetty K., Khanongnuch C. (2019). Nutritional biotransformation in traditional fermented tea (Miang) from north Thailand and its impact on antioxidant and antimicrobial activities. J. Food Sci. Technol..

[23] Chachiyo S., Kulprachakarn K., Saenjum C., Rerkasem K., Srichairatakool S., Boonyapranai K., Parklak W., Somsak V., Ounjaijean S. (2020). Toxicity evaluation of *Camellia sinensis* var. assamica and its fermented miang product. Pharmacogn. Res..

[24] Higdon J.V., Frei B. (2003). Tea catechins and polyphenols: Health effects, metabolism, and antioxidant functions. Crit. Rev. Food Sci. Nutr..

[25] Laoung-On J., Anuduang A., Saenjum C., Rerkasem K., Srichairatanakool S., Boonyapranai K., Ounjaijean S. (2025). Anti-diabetic and antioxidant effect evaluation of Thai shallot and Cha-miang in diabetic rats. Biology.

[26] Pérez-Gregorio R.M., García-Falcón M.S., Simal-Gandara J., Rodrigues A.S., Almeida D.P. (2010). Identification and quantification of flavonoids in traditional cultivars of red and white onions at harvest. J. Food Compos. Anal..

[27] Laoung-On J., Ounjaijean S., Sudwan P., Boonyapranai K. (2024). Phytochemical screening, antioxidant effect and sperm quality of the *Bomba ceiba* stamen extracts on *Charolais* cattle sperm induced by ferrous sulfate. Plants.

[28] Carrillo-Martinez E.J., Flores-Hernández F.Y., Salazar-Montes A.M., Nario-Chaidez H.F., Hernández-Ortega L.D. (2024). Quercetin, a flavonoid with great pharmacological capacity. Molecules.

[29] Bakar M.F.A., Mohamad M., Rahmat A., Burr S.A., Fry J.R. (2010). Cytotoxicity, cell cycle arrest, and apoptosis in breast cancer cell lines exposed to an extract of the seed kernel of *Mangifera pajang* (bambangan). Food Chem. Toxicol..

[30] Guillén-Meléndez G.A., Villa-Cedillo S.A., Pérez-Hernández R.A., Castillo-Velázquez U., Salas-Treviño D., Saucedo-Cárdenas O., Montes-de-Oca-Luna R., Gómez-Tristán C.A., Garza-Arredondo A.J., Zamora-Ávila D.E. (2021). Cytotoxic effect in vitro of *Acalypha monostachya* extracts over human tumor cell lines. Plants.

[31] Pham D.-C., Shibu M., Mahalakshmi B., Velmurugan B.K. (2020). Effects of phytochemicals on cellular signaling: Reviewing their recent usage approaches. Crit. Rev. Food Sci. Nutr..

[32] Ransy C., Vaz C., Lombès A., Bouillaud F. (2020). Use of H_2_O_2_ to cause oxidative stress, the catalase issue. Int. J. Mol. Sci..

[33] Purba C.C., Mayangsari Y., Setyaningsih W., Chansuwan W., Sirinupong N. (2024). Bioactive compounds of *Citrus hystrix* peel ethanolic extract and their antioxidant potential under hydrogen peroxide-induced oxidative stress in Caco-2 cells. Future Foods.

[34] Messier E.M., Bahmed K., Tuder R.M., Chu H.W., Bowler R.P., Kosmider B. (2013). Trolox contributes to Nrf2-mediated protection of human and murine primary alveolar type II cells from injury by cigarette smoke. Cell Death Dis..

[35] Kilicarslan You D., Fuwad A., Lee K.H., Kim H.K., Kang L., Kim S.M., Jeon T.-J. (2024). Evaluation of the protective role of vitamin E against ROS-driven lipid oxidation in model cell membranes. Antioxidants.

[36] Murota K., Terao J. (2003). Antioxidative flavonoid quercetin: Implication of its intestinal absorption and metabolism. Arch. Biochem. Biophys..

[37] Laoung-On J., Jitjumnong J., Sudwan P., Outaitaveep N., Ounjaijean S., Boonyapranai K. (2025). Red cotton stamen extracts mitigate ferrous sulfate-induced oxidative stress and enhance quality in bull frozen semen. Vet. Sci..

[38] Agarwal A., Virk G., Ong C., Du Plessis S.S. (2014). Effect of oxidative stress on male reproduction. World J. Mens. Health.

[39] Mukerjee S., Saeedan A.S., Ansari M.N., Singh M. (2021). Polyunsaturated fatty acids mediated regulation of membrane biochemistry and tumor cell membrane integrity. Membranes.

[40] Abd El H.A.H.M. (2012). Lipid peroxidation end-products as a key of oxidative stress: Effect of antioxidant on their production and transfer of free radicals. Lipid Peroxidation.

[41] Barros L., Cabrita L., Boas M.V., Carvalho A.M., Ferreira I.C. (2011). Chemical, biochemical and electrochemical assays to evaluate phytochemicals and antioxidant activity of wild plants. Food Chem..

[42] Yusupov M., Van der Paal J., Neyts E.C., Bogaerts A. (2017). Synergistic effect of electric field and lipid oxidation on the permeability of cell membranes. Biochim. Biophys. Acta Gen. Subj..

[43] Durlacher-Betzer K., Hassan A., Levi R., Axelrod J., Silver J., Naveh-Many T. (2018). Interleukin-6 contributes to the increase in fibroblast growth factor 23 expression in acute and chronic kidney disease. Kidney Int..

[44] Zhang X., Tian X., Wang Y., Yan Y., Wang Y., Su M., Lv H., Li K., Hao X., Xing X. (2024). Application of lipopolysaccharide in establishing inflammatory models. Int. J. Biol. Macromol..

[45] Wu J., Du X., Tong F., Zhang S., Guo J., Zhu C., Cui R., Bai Q., Chen M., Meng L. (2025). Chemical profiling, discriminant analysis, antioxidants, and anti-inflammatory activities of Lonicerae Japonicae Flos tea varieties. Future Foods.

[46] Wang Y., Wang X., Yang Y., Quan Q., Huo T., Yang S., Ju R., An Q. (2022). Comparison of the in vitro anti-inflammatory effect of cannabidiol to dexamethasone. Clin. Cosmet. Investig. Dermatol..

[47] Chojnacka K., Lewandowska U. (2023). Inhibition of pro-inflammatory cytokine secretion by polyphenol-rich extracts in macrophages via NF-κB pathway. Food Rev. Int..

[48] Xie C., Kang J., Li Z., Schauss A.G., Badger T.M., Nagarajan S., Wu T., Wu X. (2012). The açaí flavonoid velutin is a potent anti-inflammatory agent: Blockade of LPS-mediated TNF-α and IL-6 production through inhibiting NF-κB activation and MAPK pathway. J. Nutr. Biochem..

[49] Dehghan Shahreza F. (2017). Oxidative stress, free radicals, kidney disease and plant antioxidants. Immunopathol. Persa.

[50] Alsawaf S., Alnuaimi F., Afzal S., Thomas R.M., Chelakkot A.L., Ramadan W.S., Hodeify R., Matar R., Merheb M., Siddiqui S.S. (2022). Plant flavonoids on oxidative stress-mediated kidney inflammation. Biology.

